# Hyperpolarised xenon-129 diffusion-weighted magnetic resonance imaging for assessing lung microstructure in idiopathic pulmonary fibrosis

**DOI:** 10.1183/23120541.00048-2023

**Published:** 2023-08-29

**Authors:** James A. Eaden, Nicholas D. Weatherley, Ho-Fung Chan, Guilhem Collier, Graham Norquay, Andrew J. Swift, Smitha Rajaram, Laurie J. Smith, Brian J. Bartholmai, Stephen M. Bianchi, Jim M. Wild

**Affiliations:** 1 POLARIS, Department of Infection, Immunity and Cardiovascular Disease, University of Sheffield, Sheffield, UK; 2 Academic Directorate of Respiratory Medicine, Sheffield Teaching Hospitals NHS Foundation Trust, Sheffield, UK; 3 Department of Academic Radiology, University of Sheffield, Sheffield, UK; 4 Department of Radiology, Mayo Clinic, Rochester, MN, USA; 5 Insigneo Institute for In-Silico Medicine, University of Sheffield, Sheffield, UK

## Abstract

**Background:**

Hyperpolarised 129-xenon (^129^Xe) magnetic resonance imaging (MRI) shows promise in monitoring the progression of idiopathic pulmonary fibrosis (IPF) due to the lack of ionising radiation and the ability to quantify functional impairment. Diffusion-weighted (DW)-MRI with hyperpolarised gases can provide information about lung microstructure. The aims were to compare ^129^Xe DW-MRI measurements with pulmonary function tests (PFTs), and to assess whether they can detect early signs of disease progression in patients with newly diagnosed IPF.

**Methods:**

This is a prospective, single-centre, observational imaging study of patients presenting with IPF to Northern General Hospital (Sheffield, UK). Hyperpolarised ^129^Xe DW-MRI was performed at 1.5 T on a whole-body General Electric HDx scanner and PFTs were performed on the same day as the MRI scan.

**Results:**

There was an increase in global ^129^Xe apparent diffusion coefficient (ADC) between the baseline and 12-month visits (mean 0.043 cm^2^·s^−1^, 95% CI 0.040–0.047 cm^2^·s^−1^ *versus* mean 0.045 cm^2^·s^−1^, 95% CI 0.040–0.049 cm^2^·s^−1^; p=0.044; n=20), with no significant change in PFTs over the same time period. There was also an increase in ^129^Xe ADC in the lower zone (p=0.027), and an increase in ^129^Xe mean acinar dimension in the lower zone (p=0.033) between the baseline and 12-month visits. ^129^Xe DW-MRI measurements correlated strongly with diffusing capacity of the lung for carbon monoxide (% predicted), transfer coefficient of the lung for carbon monoxide (*K*_CO_) and *K*_CO_ (% predicted).

**Conclusions:**

^129^Xe DW-MRI measurements appear to be sensitive to early changes of microstructural disease that are consistent with progression in IPF at 12 months. As new drug treatments are developed, the ability to quantify subtle changes using ^129^Xe DW-MRI could be particularly valuable.

## Tweetable abstract

**Xenon-129 MRI measurements of diffusion are more sensitive to early progression of microstructural changes in IPF when compared to lung function tests, and this novel imaging method may be particularly valuable as new drug treatments are developed** https://bit.ly/3Pp6wk8

## Introduction

Idiopathic pulmonary fibrosis (IPF) is a progressive, irreversible fibrotic interstitial lung disease (ILD) of unknown aetiology. Most patients with IPF experience a steady deterioration in symptoms and pulmonary function. While some demonstrate relative stability, others die prematurely, often within the first 12 months as a result of rapidly progressive disease [1]. It has previously been suggested by an expert panel that all-cause mortality and nonelective hospitalisation are the most robust and meaningful primary end-points for IPF drug studies [2]. However, trials with all-cause mortality as the primary outcome require a large number of subjects and a long duration of follow-up with substantial associated costs, which would likely prohibit such a study from being feasible [3].

Currently, forced vital capacity (FVC) is the most recommended and validated tool to monitor the progression of IPF and is considered to be an acceptable surrogate end-point in IPF therapeutic trials, despite being relatively insensitive to longitudinal change [4], and providing no regional information about the extent of disease. We hypothesise that a new approach to investigate regional lung structure–function, which is sensitive in early disease, would be favourable.

Significant advances have been made over the past decade in the development of hyperpolarised gas magnetic resonance imaging (MRI). Due to the limited availability and increasing cost of helium (^3^He), there has been a transition to the use of 129-xenon (^129^Xe) as the preferred imaging contrast noble gas. Hyperpolarised ^129^Xe MRI shows promise as a valuable tool to monitor IPF longitudinally due to the lack of ionising radiation and the ability to quantify functional impairment [5].

Diffusion-weighted (DW) MRI with hyperpolarised gases can provide information about lung microstructure down to the alveolar level, through two approaches. First, the apparent diffusion coefficient (ADC), which is a measure of Brownian diffusion of gas atoms in the alveolar airspaces and is affected by restrictions at tissue boundaries. Secondly, complementary geometrical models can be fitted to the magnetic resonance signal behaviour, returning quantitative geometrical parameters similar to those from histology, such as the mean acinar duct radius from the “cylinder model” [6] and the mean acinar dimension (Lm_D_), from the stretched exponential model [7]. A previous DW-MRI study using hyperpolarised ^3^He in patients with IPF demonstrated that both ADC and Lm_D_ correlate with diffusing capacity of the lung for carbon monoxide (*D*_LCO_), carbon monoxide transfer coefficient (*K*_CO_) and regional fibrosis on high-resolution computed tomography (HRCT) [8]. There was no significant longitudinal change in ADC, FVC or *D*_LCO_, although Lm_D_ increased significantly over 12 months (p=0.001). To date, no data are available on the utility of ^129^Xe DW-MRI in IPF. The aims of this study were to compare ^129^Xe DW-MRI measurements with pulmonary function tests (PFTs), and to assess whether ^129^Xe DW-MRI measurements can detect early signs of disease progression in newly diagnosed IPF patients.

## Methods

This is a prospective, single-centre, observational imaging study of patients presenting with IPF to Northern General Hospital (Sheffield, UK). The diagnosis of IPF was based on the most recent official American Thoracic Society/European Respiratory Society/Japanese Respiratory Society/Latin American Thoracic Society clinical practice guideline for the diagnosis of IPF [9] and established in ILD multidisciplinary team meetings involving respiratory physicians and thoracic radiologists. The study was given ethical approval by the North West-Liverpool Central NHS research ethics committee under reference 15/NW/0750. It was sponsored by Sheffield Teaching Hospitals Research and Development (STH18876) and was registered with the NIHR Clinical Research Network Portfolio, with UKCRN ID 20468. Written consent was provided by all subjects. Data presented is from the same study protocol as published previously [8], but from a different patient cohort, and importantly using ^129^Xe DW-MRI instead of ^3^He DW-MRI.

The inclusion criteria included diagnosis of IPF (as determined by the Sheffield ILD multidisciplinary team) within 1 year, oxygen saturations ≥90% in room air and age 18–80 years. The exclusion criteria included patients on immunosuppressive treatment (excluding prednisolone at a dose of ≤20 mg·day^−1^ or N-acetylcysteine), pregnancy, renal impairment (glomerular filtration rate <30 mL·min^−1^), oxygen saturations <90% in room air, age >80 years or <18 years at the onset of the study, inability to lie supine comfortably for ≥60 min, significant comorbidity likely to reduce life expectancy to <1 year, severe ischaemic heart disease or symptoms of angina not fully controlled, significant congestive cardiac failure, any contraindication(s) to MRI scanning and previous allergy to MRI contrast agent (gadolinium). All PFTs were performed on the same day as the MRI scans. The Global Lung Initiative 2012 reference equations [10] were used to calculate the % predicted values.

### HRCT acquisition and analysis

The computed tomography (CT) protocol (unenhanced volumetric HRCT thorax with full lung coverage) used was standardised for each scan. Acquisition details: scan mode=helical, rotation time=0.5 s, total scan time=5.8 s, X-ray beam filter=UE0, lung inflation state=total lung capacity. Reconstruction details: slice thickness=1.0 mm, slice interval=1.0 mm, reconstruction process=Advanced intelligent Clear-IQ Engine Lung Standard, reconstruction field of view=400.4 (L). HRCT scans were performed on various scanners (mainly Siemens or Toshiba).

The CT scan which was used in the ILD multidisciplinary team meeting for diagnostic purposes was used as the baseline CT scan in the study. Therefore, a baseline HRCT scan was not acquired routinely for research purposes unless the diagnostic CT scan was not a volumetric inspiratory noncontrast HRCT scan. As a result, there was a period of up to 12 months between the baseline HRCT scan and the first study visit. The mean time between the baseline HRCT and the first study visit was 143 days.

Quantitative CT analysis was performed on all scans using Computer-Aided Lung Informatics for Pathology Evaluation and Rating (CALIPER) software at the Mayo Clinic (Rochester, MA, USA). The CALIPER data included volumetric parenchymal pattern classification of each pixel into ground-glass opacity (GGO), low-attenuation areas (*e.g.* emphysema), honeycombing, reticulation or normal tissue [11]. The global percentage of each of these parenchymal patterns was calculated by dividing the corresponding volume by the total lung volume. Honeycombing % and reticulation % were combined and identified as fibrosis %. GGO %, honeycombing % and reticulation % were combined and identified as ILD %. The CALIPER software performs automated segmentation of the vessel-related structures in the lung excluding the large vessels at the hilum [12]. In addition, regional data were collected, as the CALIPER software can classify the same volumetric data by distribution in the lungs, thereby producing analysis of upper, middle and lower zones in each lung. The upper zone was classified as the region above the carina, and the middle/lower zones were based on 50% craniocaudal distance between the carina and the most inferior extent of the lungs. Upper, middle and lower zone % values were produced by adding the right and left lung values together for each of the corresponding zones. A semi-quantitative visual CT scoring system (table 1) was used by two experienced consultant chest radiologists in Sheffield. Honeycombing and reticulation were combined and identified as fibrosis. GGO, honeycombing and reticulation were combined and identified as ILD.

**TABLE 1:** Semi-quantitative visual computed tomography scoring system

**Abnormality**	**Grading for each abnormality**		**Anatomical regions scored**
	**Disease extent %**	**Score**	
**GGO alone**	0	0	Lobes are scored independently Lingula is considered a separate lobe Global score: summation of scores for each abnormality, in all lobes
**Mixed GGO and reticular disease**	1–25	1	
**Reticular fibrosis alone**	26–50	2	
**Honeycombing**	51–75	3	
**Consolidation**	>75	4	

GGO: ground-glass opacity.

### ^129^Xe DW-MRI protocol

All pulmonary MRI was performed at 1.5 T on a whole-body General Electric HDx scanner at the University of Sheffield MRI department. ^129^Xe was polarised on site under regulatory licence to >30% using a custom-made spin exchange optical polariser capable of generating 550-mL doses in <10 min [13]. All ^129^Xe images were acquired at functional residual capacity (FRC) +1 L in a flexible quadrature transmit/receive coil (Clinical MR solutions, Brookfield, WI, USA). Continuous monitoring of the patient's heart rate and oxygen saturations were performed during the ^129^Xe MRI scans.

DW-MRI was performed using a three-dimensional spoiled gradient echo multiple b-value sequence (b=0, 12, 20, 30 s·cm^−2^) with compressed sensing [14]. Subjects inhaled a mixture of 550 mL ^129^Xe and 450 mL nitrogen from FRC and the breath-hold time was 16 s. Maps of ^129^Xe ADC and Lm_D_ were calculated for each imaging voxel using a mono-exponential fit to data at the first two DW b-values (b=0, 12 s·cm^−2^), and a stretched-exponential fit to data at all b-values, respectively [7]. Global and regional values (upper/middle/lower zones of the lungs) were calculated. Each of the upper, middle and lower zones corresponded to approximately a third of the total lung height. Data analysis was performed using in-house MATLAB (MathWorks, Natick, MA, USA) code at the University of Sheffield.

### Statistics

Continuous variables were stated as mean or median as appropriate according to the distribution of data. The Pearson correlation coefficient (nonparametric data: Spearman's rank correlation) was used to determine the strength of associations. Paired data were analysed for variance using the paired-sample t-test (nonparametric data: Wilcoxon paired-sample test) for comparisons over two time points. Statistical tests of normality were performed on each set of data to determine the most appropriate statistical test. All tests were two-tailed and statistical significance was assumed at p<0.05. Data were analysed using GraphPad Prism (San Diego, CA, USA) version 9.0.1.

## Results

### Subject demographics

34 patients with IPF (diagnosed within 12 months) were recruited into the study between May 2017 and November 2019. Three subjects did not tolerate the baseline MRI scan and did not complete the baseline study visit; therefore, their data were not included in the results. 20 subjects returned at 12 months (mean 421 days). Of the 14 subjects who did not attend the 12-month follow-up visit, four had died within the 12-month follow-up period. The remaining 10 subjects withdrew from the study, with the main reason being difficulty tolerating the baseline MRI scan. Due to coronavirus disease 2019 (COVID-19) restrictions, seven of the 12-month study visits were delayed for 2–5 months. Once the impact of COVID-19 on the study was realised, the study protocol was amended and subsequently approved to allow the 12-month study visit to take place up to 18 months post-baseline visit. The mean time between the baseline study visit and the 12-month study visit was 421 days.

The baseline demographic data for the study cohort (mean±sd) are as follows: age 69.9±11.6 years, male 77.4%, ever-smoker 51.6%, body mass index (BMI) 28.0±4.22 kg·m^−2^.

### Pulmonary function tests

PFTs at baseline and for the cohort attending both the baseline and 12-month visits are presented in tables 2 and 3, respectively.

**TABLE 2:** Pulmonary function test results at the baseline visit (n=31)

**Spirometry**	
FEV_1_ (L)	2.44±0.53
FEV_1_ (% predicted)	88.6±15.1
FVC (L)	3.28±0.76
FVC (% predicted)	90.5±16.1
**Gas exchange tests**	
*D*_LCO_ (mmol·min^−1^·kPa^−1^)	5.36±1.60
*D*_LCO_ (% predicted)	69.2±16.3
*K*_CO_ (mmol·min^−1^·kPa^−1^·L^−1^)	1.16±0.24
*K*_CO_ (% predicted)	84.8±17.8

Data are presented as mean±sd. FEV_1_: forced expiratory volume in 1 s; FVC: forced vital capacity; *D*_LCO_: diffusing capacity of the lung for carbon monoxide; *K*_CO_: carbon monoxide transfer coefficient.

**TABLE 3:** Pulmonary function test results for the cohort attending both the baseline and 12-month visits (n=20)

	**Baseline visit**	**12-month visit**	**p-value**
**FEV_1_ (L)**	2.50 (2.24–2.77)	2.47 (2.25–2.68)	0.58
**FEV_1_ (% predicted)**	90.2 (81.9–98.4)	89.9 (83.4–96.5)	0.93
**FVC (L)**	3.42 (3.03–3.81)	3.36 (3.01–3.70)	0.54
**FVC (% predicted)**	93.3 (85.3–101.4)	92.8 (85.2–100.5)	0.86
***D*_LCO_ (mmol·min^−1^·kPa^−1^)**	5.61 (4.82–6.40)	5.40 (4.50–6.31)	0.20
***D*_LCO_ (% predicted)**	71.6 (64.1–79.2)	69.6 (59.8–79.3)	0.34
***K*_CO_ (mmol·min^−1^·kPa^−1^·L^−1^)**	1.19 (1.07–1.31)	1.17 (1.02–1.31)	0.23
***K*_CO_ (% predicted)**	86.6 (77.8–95.3)	84.8 (74.5–95.0)	0.27

Data are presented as mean (95% CI), unless otherwise stated. FEV_1_: forced expiratory volume in 1 s; FVC: forced vital capacity; *D*_LCO_: diffusing capacity of the lung for carbon monoxide; *K*_CO_: carbon monoxide transfer coefficient.

### ^129^Xe DW-MRI

**FIGURE 1:**
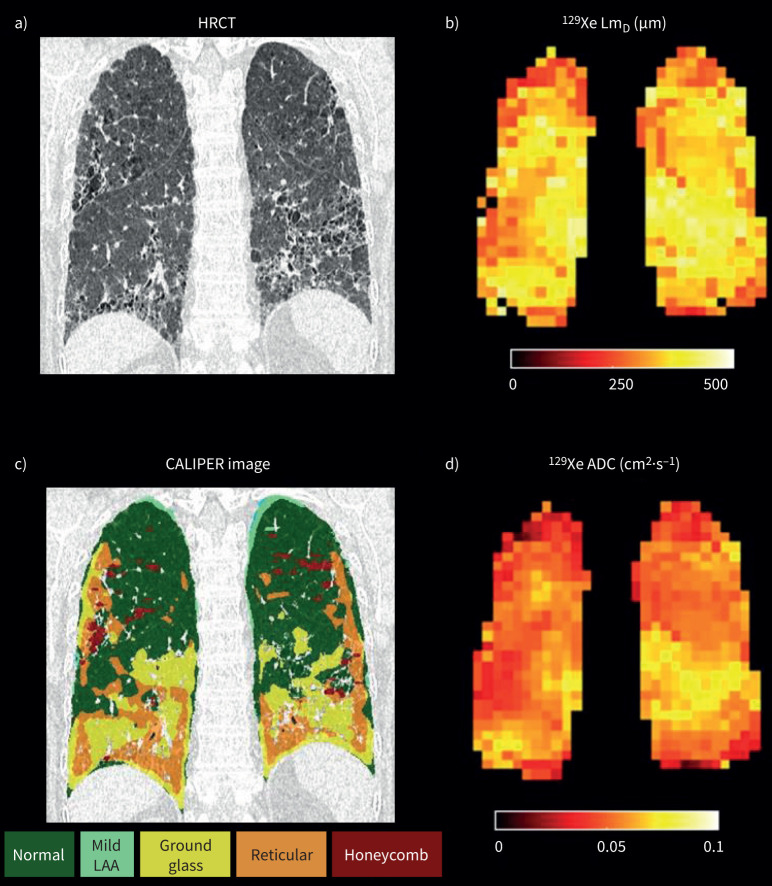
a) High-resolution computed tomography (HRCT) image, b) 129-xenon (^129^Xe) mean acinar dimension (Lm_D_) map, c) Computer-Aided Lung Informatics for Pathology Evaluation and Rating (CALIPER) image and d) ^129^Xe apparent diffusion coefficient (ADC) map in an idiopathic pulmonary fibrosis subject. LAA: low-attenuation area.

Figure 1 compares a HRCT image, CALIPER image, ^129^Xe Lm_D_ map and ^129^Xe ADC map from one of the subjects in the study.

**FIGURE 2:**
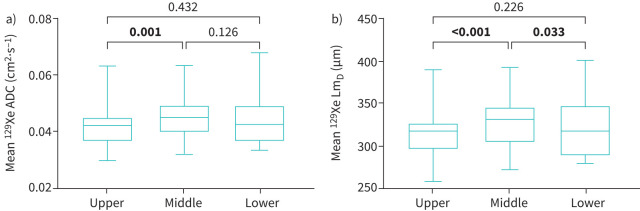
129-Xenon (^129^Xe) a) apparent diffusion coefficient (ADC) and b) mean acinar dimension (Lm_D_) in the upper, middle and lower zones at the baseline visit (n=31). Bold type represents statistical significance.

The baseline visit, mean±sd^129^Xe DW-MRI results are as follows: ADC 0.044±0.007 cm^2^·s^−1^, Lm_D_ 324±27.4 µm. At the baseline visit, there was a significant difference between the upper, middle and lower zones in ^129^Xe ADC (p=0.002; upper *versus* middle zone p=0.001; n=31) and ^129^Xe Lm_D_ (p<0.001; upper *versus* middle zone p<0.001; middle *versus* lower zone p=0.033; n=31) (figure 2a, b, table 4).

**TABLE 4:** Regional 129-xenon (^129^Xe) apparent diffusion coefficient (ADC) and mean acinar dimension (Lm_D_) results at the baseline visit (n=31)

	**^129^Xe ADC (cm^2^·s^−1^)**	**^129^Xe Lm_D_ (µm)**
**Upper zone**	0.042 (0.037–0.045)	318 (296–327)
**Middle zone**	0.045 (0.040–0.049)	332 (305–346)
**Lower zone**	0.043 (0.037–0.049)	318 (290–348)

Data are presented as median (interquartile range).

**FIGURE 3:**
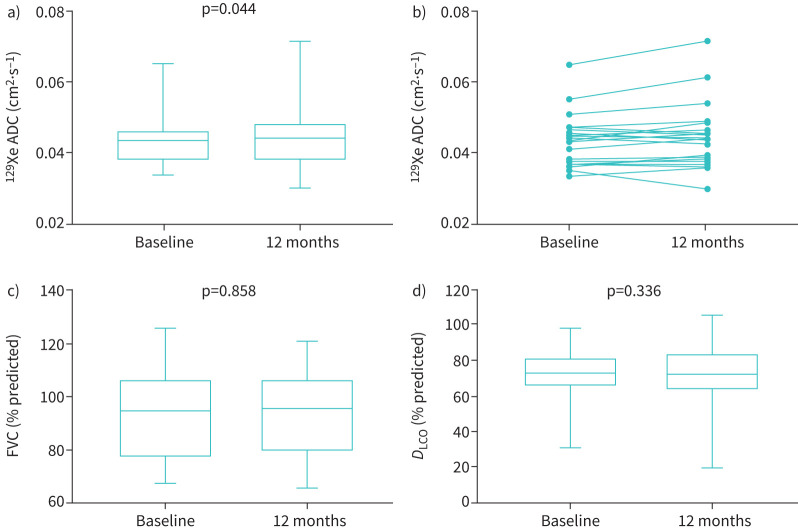
a, b) 129-Xenon (^129^Xe) apparent diffusion coefficient (ADC), c) forced vital capacity (FVC) % predicted and d) diffusing capacity of the lung for carbon monoxide (*D*_LCO_) % predicted at the baseline *versus* 12-month visit (n=20).

There was an increase in global ^129^Xe ADC between the baseline and 12-month visits (mean 0.043 cm^2^·s^−1^, 95% CI 0.040–0.047 cm^2^·s^−1^ *versus* 0.045 cm^2^·s^−1^, 95% CI 0.040–0.049 cm^2^·s^−1^; p=0.044; n=20), with no significant change in PFTs over the same time period (figure 3). However, there was no significant change in global ^129^Xe Lm_D_ between the baseline and 12-month visits (mean 320 µm, 95% CI 306–334 µm *versus* 325 µm, 95% CI 308–342 µm; p=0.085; n=20).

An increase in ^129^Xe ADC was found between the baseline and 12-month visits in the lower zone (median 0.039 cm^2^·s^−1^, 95% CI 0.035–0.044 cm^2^·s^−1^ *versus* 0.043 cm^2^·s^−1^, 95% CI 0.037–0.049 cm^2^·s^−1^; p=0.027; n=20). There was also an increase in ^129^Xe Lm_D_ between the baseline and 12-month visits in the lower zone (median 302 µm, 95% CI 285–319 µm *versus* 320 µm, 95% CI 300–340 µm; p=0.033; n=20) (table 5).

**TABLE 5:** Regional 129-xenon (^129^Xe) apparent diffusion coefficient (ADC) and mean acinar dimension (Lm_D_) results at the baseline and 12-month visits (n=20)

	**Baseline visit**	**12-month visit**	**p-value**
**^129^Xe ADC (cm^2^·s^−1^)**			
Upper zone	0.042 (0.038–0.045)	0.042 (0.038–0.046)	0.64
Middle zone	0.044 (0.041–0.048)	0.045 (0.041–0.050)	0.11
Lower zone	0.039 (0.035–0.044)	0.043 (0.037–0.049)	0.027
**^129^Xe Lm_D_ (µm)**			
Upper zone	313 (298–328)	314 (298–331)	0.68
Middle zone	326 (312–339)	330 (313–346)	0.22
Lower zone	302 (285–319)	320 (300–340)	0.033

Data are presented as median (95% CI).

### Correlations

At the baseline visit, ^129^Xe ADC was strongly correlated with *D*_LCO_ % predicted (r=−0.65; p<0.001), *K*_CO_ (r=−0.73; p<0.001) and *K*_CO_ % predicted (r=−0.68; p<0.001), and ^129^Xe Lm_D_ was also strongly correlated with *D*_LCO_ % predicted (r=−0.66; p<0.001), *K*_CO_ (r=−0.74; p<0.001) and *K*_CO_ % predicted (r=−0.69; p<0.001).

Between the baseline and 12-month visits, a strong correlation was seen between the change in global ^129^Xe ADC and *K*_CO_ (r=−0.64; p=0.002) and between the change in global ^129^Xe Lm_D_ and *K*_CO_ (r=−0.62; p=0.004). There was a moderate correlation between the change in global ^129^Xe ADC and *D*_LCO_ (r=−0.50; p=0.026) over 12 months.

At the baseline visit in the lower zone, ^129^Xe ADC was moderately correlated with semi-quantitative visual CT fibrosis score (r=0.53; p=0.002), honeycomb score (r=0.42; p=0.018) and ILD score (r=0.55; p=0.001), and ^129^Xe Lm_D_ was also moderately correlated with visual CT fibrosis score (r=0.52; p=0.003), honeycomb score (r=0.45; p=0.011) and ILD score (r=0.55; p=0.001). At the baseline visit, there was no correlation between the ^129^Xe DW-MRI measurements and any of the quantitative CALIPER CT measurements.

## Discussion

In this study, there was an increase in global ^129^Xe ADC between the baseline and 12-month visits, with no significant change in PFTs over the same time period. There was also an increase in ^129^Xe ADC and ^129^Xe Lm_D_ in the lower zone between the baseline and 12-month visits. These results suggest that ^129^Xe DW-MRI measurements are more sensitive to early progression of microstructural changes in IPF when compared to PFTs and build upon previous ^3^He DW-MRI findings [8]. As new drug treatments are developed, the ability to quantify subtle changes using ^129^Xe DW-MRI measurements could be particularly valuable.

IPF is characterised by a histopathological and radiological pattern of usual interstitial pneumonia, with fibrotic changes typically located in the subpleural areas of the lower lobes. Therefore, one would expect ^129^Xe DW-MRI measurements to be higher in the lower zone when compared to the middle zone. However, as demonstrated in table 4, this was not the case. The reason for this is probably because the software used for analysis allows segmentation of the bronchi, but segmentation of the remaining smaller conducting airways is not possible due to limitations in spatial resolution in the DW-MRI. Therefore, some of the larger more proximal airways will have contributed to the higher ^129^Xe DW-MRI measurements found in the middle zones at the baseline study visit. In previous work we have found cardiogenic modulation of the lung ventilation signal and we acknowledge the same effects coupled with cardiac motion could cause some uncertainty in the ^129^Xe DW-MRI metrics in the lower left lung.

It is likely that in IPF patients, increased ^129^Xe ADC and Lm_D_ is a result of reduced acinar integrity as a consequence of fibrotic changes in the lung, such as honeycombing and possibly traction bronchiectasis. This is supported by the findings of a correlation between ^129^Xe DW-MRI measurements and visual CT fibrosis score, honeycomb score and ILD score in the lower zone.

^129^Xe DW-MRI measurements correlated strongly with *D*_LCO_ % predicted, *K*_CO_ and *K*_CO_ % predicted. These correlations suggest that the elevated ^129^Xe DW-MRI measurements are associated with decreased gas exchange in the alveoli due to a reduction in the alveolar surface area and are in keeping with correlations seen between ^3^He DW-MRI measurements and the gas exchange measurements *D*_LCO_ and *K*_CO_ [8]. It is unclear why there was no correlation between the ^129^Xe DW-MRI measurements and any of the quantitative CALIPER CT measurements. However, it may be due to the subjects having relatively mild disease, or a discrepancy between the CALIPER CT analysis and the radiologist's visual CT scoring.

The main limitation of this work is the relatively small number of subjects in the study, which limits the inferences. However, the number of subjects in this cohort is larger than in previously published ^129^Xe MRI studies in IPF [5, 15–21]. In 10 subjects, COVID-19 restrictions led to a delay of up to 6 months in the 12-month visit, which may have affected the results of longitudinal change in biomarkers between the baseline and 12-month visits.

The ^129^Xe MRI sequences involve breath-hold manoeuvres performed at FRC plus 1 L of gas, although there is likely to be a degree of variability due to lack of spirometric control. In theory, this could alter the alveolar inflation, which could in turn affect DW-MRI measurements. However, this is unlikely to be significant, especially given the high degree of same-day reproducibility previously demonstrated with the use of ^3^He DW-MRI in IPF [8]. Data from 10 healthy volunteers at our centre using the same MRI protocols has also shown excellent repeatability in ^129^Xe ADC (intraclass correlation coefficient (ICC) 0.98) and ^129^Xe Lm_D_ (ICC 0.97) [22]. Furthermore, comparable repeatability of ^129^Xe ADC (ICC 0.94) has been reported in patients with COPD [23].

Four subjects were already taking antifibrotic therapy at the time of recruitment and one subject commenced nintedanib after the baseline study visit. It is difficult to determine whether antifibrotic therapy influenced the longitudinal changes in biomarkers due to the small sample size. It would be interesting to incorporate the MRI and quantitative CT biomarkers into future randomised antifibrotic trials to investigate the treatment effects in IPF.

The findings reported in this study represent the first known longitudinal data of hyperpolarised ^129^Xe DW-MRI measurements alongside PFTs and quantitative CT in IPF. It is anticipated that hyperpolarised ^129^Xe DW-MRI alongside dissolved ^129^Xe MRI metrics of gas transfer [5, 15] could play an important future role in the development of objective, reproducible and sensitive imaging biomarkers for the monitoring of IPF progression and assessment of response to treatment. However, before these imaging biomarkers can be implemented into routine clinical practice, they will require technical validation and will likely need to demonstrate utility as surrogate end-points in future multicentre IPF drug trials.
